# Against All Odds: Treating Septic Shock and Massive Hemorrhage in a Patient With Placenta Accreta

**DOI:** 10.7759/cureus.88938

**Published:** 2025-07-28

**Authors:** Yasmine Habli, Alice O'Brien, Jennifer Hoayek

**Affiliations:** 1 Department of Anesthesiology, Critical Care and Pain Medicine, University of Texas Health Science Center at Houston, Houston, USA

**Keywords:** cesarean hysterectomy, massive hemorrhage, maternal morbidity, obstetric anesthesia, placenta accreta spectrum (pas)

## Abstract

Placenta accreta spectrum (PAS), maternal sepsis, and hemorrhagic shock remain significant contributors to maternal morbidity and mortality. We present the case of a 36-year-old female with placenta accreta and preterm premature rupture of membranes (PPROM) who developed septic shock and underwent an emergent cesarean hysterectomy at 28 weeks of gestation. Her intraoperative course was complicated by massive hemorrhage with an estimated blood loss of 40 liters, cardiac arrest, and disseminated intravascular coagulation (DIC). Return of spontaneous circulation was achieved, and she underwent extensive surgical intervention followed by recovery in the ICU. This report underscores the vital importance of early recognition, aggressive resuscitation, and multidisciplinary coordination in managing patients with PAS, particularly when complicated by multifactorial shock. It also highlights the need for institutional readiness and improved sepsis screening tools tailored to the unique physiology of pregnancy.

## Introduction

Postpartum hemorrhage (PPH) and maternal sepsis are among the leading causes of maternal and fetal morbidity and mortality in the United States, according to the most recent data from the Maternal Mortality Review Committees of the Centers for Disease Control (CDC) [1,2]. In pregnancies complicated by abnormal placentation, such as placenta accreta spectrum (PAS), the risk of PPH increases significantly, often necessitating complex surgical intervention, such as cesarean hysterectomy (C-hyst), which further increases the risk of complications and maternal morbidity [3]. Furthermore, the physiological changes of pregnancy can mask early signs of sepsis, leading to a delay in diagnosis and intervention [4]. Preterm premature rupture of membranes (PPROM) occurs in approximately 2-4% of singleton pregnancies [5]. It is associated with increased risk of cesarean delivery, chorioamnionitis, prolonged hospitalization, and the need for neonatal intensive care, all of which may increase the risk for both PPH and sepsis in affected patients [6]. We present the case of a 36-year-old patient with PAS and PPROM who underwent emergent C-hyst complicated by septic shock, hypovolemic shock due to massive hemorrhage, and cardiac arrest.

This case was previously presented as a meeting abstract at the 2024 Society for Obstetric Anesthesia and Perinatology (SOAP) Annual Scientific Meeting on May 2, 2024.

## Case presentation

A 36-year-old female (gravida 4 para 3) with a history of three prior cesarean deliveries presented to our institution with PPROM at 26 weeks of gestation and was diagnosed with placenta accreta and a short cervix. She was admitted for observation with a plan for a C-hyst at 34 weeks of gestational age (GA). However, on hospital day 15, at 28 weeks and two days of GA, the patient developed significant tachycardia (heart rate of 170 beats per minute), hypotension (mean arterial pressures of 40-50 mmHg), fever, chills, and shortness of breath in the antepartum unit. Due to a non-reassuring fetal heart rate of 60 beats per minute, the patient was rushed to the operating room for a STAT cesarean delivery. She underwent general anesthesia, and phenylephrine and norepinephrine infusions were immediately initiated to maintain mean arterial pressures above 65 mmHg. A radial arterial line was placed for hemodynamic monitoring immediately after induction.

Two 14- and 16-gauge (G) intravenous (IV) catheters were inserted in the antecubital right and left arms and used for resuscitation along with a pre-existing peripherally inserted central catheter (PICC) line and a 20 G peripheral IV line. Laboratory work obtained immediately before the patient was taken to the OR showed a lactic acid of 14.9 mmol/l (reference range: 0.5-2/2 mmol/l), white cell count of 5,800 (reference range: 4,150-10,550 /uL), hemoglobin of 12.5 g/dL (reference range 10.8-14.8 g/dL) and hematocrit of 38.5% (reference range: 33.9-45.4%). Septic shock was suspected, and empiric broad-spectrum antibiotics (cefazolin, vancomycin, and metronidazole) were initiated rapidly intraoperatively. The baby was delivered within five minutes of the induction of anesthesia. Following delivery, significant bleeding was noted, prompting activation of the Massive Transfusion Protocol (MTP).

The intraoperative course was complicated by uncontrollable refractory hemorrhage at the surgical site, culminating in cardiac arrest secondary to hypovolemic shock. Return of spontaneous circulation was achieved following one cycle of cardiopulmonary resuscitation with 1 mg IV of epinephrine, followed by defibrillation of ventricular tachycardia, which converted to sinus rhythm. The remainder of the intraoperative course was complicated due to the patient developing disseminated intravascular coagulation (DIC), which made achieving hemostasis difficult. She also continued to be hypotensive, requiring vasopressors from a combination of sepsis and ongoing hemorrhage from friable tissue associated with placenta accreta. Correction of DIC was achieved with targeted transfusion of blood products and clotting factors guided by thromboelastography (TEG) alongside successful surgical efforts to control the bleeding.

The patient underwent a hysterectomy, bilateral salpingectomy, left oophorectomy, iatrogenic cystotomy repair, and bilateral ligation of the hypogastric artery. The total surgical duration was approximately six hours from incision to closure. The estimated blood loss was 40,478 ml. The patient received a total of 63 units of packed red blood cells (RBCs), 66 units of fresh frozen plasma (FFP), nine jumbo platelets (1776 ml), six units of cryoprecipitate, and 1.5 liters of 5% albumin. Additionally, she was given a total of two grams of IV tranexamic acid and multiple doses of calcium chloride. Urine output was continuously monitored and was recorded at 1,300 mL over the course of the procedure. She was maintained on vasopressor support with phenylephrine, norepinephrine, and vasopressin throughout until the bleeding was effectively controlled. The abdomen was packed, and the patient was transferred to the ICU, intubated, sedated, off vasopressor infusions, and in stable condition. Following appropriate intraoperative resuscitation, her post-hysterectomy hemoglobin was 8.2 g/dl with correction and normalization of both coagulation and TEG studies.

On postoperative day (POD) one, she was taken back to the operating room for re-exploration and closure of the abdomen. On POD two, the patient was successfully extubated, and on the third POD, she was deemed stable for transfer out of the ICU to the Labor and Delivery unit. During her ICU stay, the patient required additional transfusions with four units of PRBC and three units of FFP. The baby was admitted to the neonatal ICU after delivery in stable condition.

## Discussion

This case highlights the complexity and challenges associated with managing PAS in the setting of PPROM, further complicated by septic shock, DIC, and massive hemorrhage. The extraordinary scale of complications encountered in this case, including a total estimated blood loss of 40 liters and the transfusion of more than 140 units of blood products, underscores the critical importance of rapid recognition and timely intervention, in addition to the implementation of well-established institutional protocols. To our knowledge, bleeding of this magnitude is extremely rare, and cases of multifactorial shock in this clinical context have not been previously reported in the literature. C-hyst remains the standard approach for managing PAS. Its effective intraoperative management requires vigilant monitoring of hemodynamics, volume status, urine output, and blood loss, in conjunction with effective interdisciplinary coordination. This helps to ensure real-time awareness of the patient’s condition and guide timely interventions and anticipate transfusion requirements [3].

In our case, sepsis secondary to chorioamnionitis introduced additional challenges, further exacerbating hemodynamic instability and contributing to significant coagulopathy. DIC, reported in up to 35% of severe sepsis cases and associated with increased maternal morbidity [7], significantly exacerbated the clinical picture and complicated efforts to achieve hemostasis. The surgical complexity associated with PAS, combined with DIC and sepsis, resulted in substantial quantitative blood loss and transfusion volumes significantly exceeding those typically reported for a C-hyst. In comparison, a study from the UK found that women who underwent peripartum hysterectomy had a median transfusion requirement of 10 units of packed red cells and four units of FFP [8]. Our patient presented with multiple risk factors for PPH, including PAS, emergent cesarean delivery, chorioamnionitis complicated by sepsis, and peripartum hysterectomy; collectively creating a ‘perfect storm’ resulting in severe hemorrhagic complications. Notably, each of these factors is independently associated with an increased risk of PPH [9]. Additionally, the emergent nature of the surgery further contributed to the morbidity risk, as a planned C-hyst compared to an emergent C-hyst has been associated with reduced transfusion requirements and improved neonatal outcomes [10].

This case offers several important lessons for clinical practice. Early activation of MTP is crucial, as it has been shown to improve survival in cases of severe PPH [11]. Timely access to a high-functioning blood bank and the availability of trained personnel familiar with MTP protocols are fundamental in caring for high-risk parturients such as our patient. In this case, the rapid provision of blood products combined with the coordinated efforts of a well-trained multidisciplinary team (including maternal-fetal medicine specialists, obstetricians, anesthesiologists, neonatologists, surgical consultants, critical care specialists, and experienced nursing staff) was pivotal in managing the massive hemorrhage and ensuring positive maternal and fetal outcomes. Nonetheless, significant maternal morbidity may still occur even under optimal conditions [3]. Additionally, this case highlights the importance of early recognition and aggressive treatment of infection in high-risk pregnancies. Maternal sepsis contributes to coagulopathy and increased tissue friability, making surgical intervention and hemorrhage control more challenging. Notably, chorioamnionitis has been associated with an increased risk of hemorrhage in cesarean deliveries [9].

Despite the favorable maternal and neonatal outcomes in this case, some limitations warrant consideration. The physiological changes of pregnancy, such as elevated heart rate and decreased systemic vascular resistance, can mask early signs of sepsis, which may cause a delay in diagnosis and intervention. Early hemorrhagic shock can closely mimic septic shock, complicating timely recognition and management. Given that PPROM is associated with an increased risk of cesarean delivery, chorioamnionitis, prolonged hospitalization, and neonatal intensive care admission [6], it is essential to maintain a high index of suspicion for any signs of infection. However, conventional sepsis scoring systems fail to account for physiological changes of pregnancy, limiting their reliability in the obstetric population. To address this limitation, researchers have proposed specialized indices to help identify severe infections in parturients. For example, obstetric and sepsis-specific early warning systems (EWS) have demonstrated superior sensitivity in detecting maternal sepsis compared to general scoring systems. However, their effectiveness in real-world settings is often hindered by incomplete clinical data [12].

## Conclusions

Early identification and a coordinated multidisciplinary approach are essential in managing high-risk pregnancies complicated by PPROM, infection, and hemorrhage. This report emphasizes the importance of institutional preparedness, multidisciplinary team training, and the implementation of coordinated protocols for effective management of both hemorrhage and sepsis. Advancing maternal care will require future research focused on refining predictive tools, improving resource allocation during obstetric emergencies, and developing and validating early sepsis detection tools and multidisciplinary response protocols for this unique patient population.

## References

[1] (2025). CDC: pregnancy-related deaths: data from Maternal Mortality Review Committees in 36 U.S. States, 2017-2019. Maternal mortality prevention. https://www.cdc.gov/maternal-mortality/php/data-research/mmrc-2017-2019.html.

[2] (2025). CDC: pregnancy-related deaths: data from Maternal Mortality Review Committees in 38 U.S. States, 2020. Maternal mortality prevention. States.

[3] Cahill AG, Beigi R, Heine RP, Silver RM, Wax JR (2018). Placenta accreta spectrum. Am J Obstet Gynecol.

[4] Escobar MF, Echavarría MP, Zambrano MA, Ramos I, Kusanovic JP (2020). Maternal sepsis. Am J Obstet Gynecol MFM.

[5] (2016). Practice Bulletin no. 160: premature rupture of membranes. Obstet Gynecol.

[6] Wahabi H, Elmorshedy H, Bakhsh H (2024). Predictors and outcomes of premature rupture of membranes among pregnant women admitted to a teaching Hospital in Saudi Arabia: a cohort study. BMC Pregnancy Childbirth.

[7] Levi M, Scully M (2018). How I treat disseminated intravascular coagulation. Blood.

[8] Knight M (2007). Peripartum hysterectomy in the UK: management and outcomes of the associated haemorrhage. BJOG.

[9] Mark SP, Croughan-Minihane MS, Kilpatrick SJ (2000). Chorioamnionitis and uterine function. Obstet Gynecol.

[10] Thang NM, Anh NT, Thanh PH, Linh PT, Cuong TD (2021). Emergent versus planned delivery in patients with placenta accreta spectrum disorders: a retrospective study. Medicine (Baltimore).

[11] Massoth C, Wenk M, Meybohm P, Kranke P (2023). Coagulation management and transfusion in massive postpartum hemorrhage. Curr Opin Anaesthesiol.

[12] O'Regan C, O'Malley EG, Power KA, Reynolds CM, Sheehan SR, Turner MJ (2019). Development of a novel bedside index for the early identification of severe maternal infection. Eur J Obstet Gynecol Reprod Biol.

